# Concentration of endogenous estrogens and estrogen metabolites in the NCI-60 human tumor cell lines

**DOI:** 10.1186/gm330

**Published:** 2012-04-30

**Authors:** Xia Xu, Timothy D Veenstra

**Affiliations:** 1 Laboratory of Proteomics and Analytical Technologies, SAIC-Frederick, Inc., National Cancer Institute at Frederick, Frederick, MD 21702, USA

## Abstract

**Background:**

Endogenous estrogens and estrogen metabolites play an important role in the pathogenesis and development of human breast, endometrial, and ovarian cancers. Increasing evidence also supports their involvement in the development of certain lung, colon and prostate cancers.

**Methods:**

In this study we systemically surveyed endogenous estrogen and estrogen metabolite levels in each of the NCI-60 human tumor cell lines, which include human breast, central nerve system, colon, ovarian, prostate, kidney and non-small cell lung cancers, as well as melanomas and leukemia. The absolute abundances of these metabolites were measured using a liquid chromatography-tandem mass spectrometry method that has been previously utilized for biological fluids such as serum and urine.

**Results:**

Endogenous estrogens and estrogen metabolites were found in all NCI-60 human tumor cell lines and some were substantially elevated and exceeded the levels found in well known estrogen-dependent and estrogen receptor-positive tumor cells such as MCF-7 and T-47D. While estrogens were expected to be present at high levels in cell lines representing the female reproductive system (that is, breast and ovarian), other cell lines, such as leukemia and colon, also contained very high levels of these steroid hormones. The leukemia cell line RMPI-8226 contained the highest levels of estrone (182.06 pg/10^6 ^cells) and 17β-estradiol (753.45 pg/10^6 ^cells). In comparison, the ovarian cancer cell line with the highest levels of these estrogens contained only 19.79 and 139.32 pg/10^6 ^cells of estrone and 17β-estradiol, respectively. The highest levels of estrone and 17β-estradiol in breast cancer cell lines were only 8.45 and 87.37 pg/10^6 ^cells in BT-549 and T-47D cells, respectively.

**Conclusions:**

The data provided evidence for the presence of significant amounts of endogenous estrogens and estrogen metabolites in cell lines not commonly associated with these steroid hormones. This broad discovery of endogenous estrogens and estrogen metabolites in these cell lines suggest that several human tumors may be beneficially treated using endocrine therapy aimed at estrogen biosynthesis and estrogen-related signaling pathways.

## Background

Endogenous estrogens and estrogen metabolites (EMs) have long been associated with carcinogenesis and development of several hormone-dependent human carcinomas, such as breast, endometrial, and ovarian cancers [1,2]. Increasing evidence suggests that these metabolites may be involved in the pathogenesis and development of human lung [3,4] and colon [5] cancers as well as prostate cancer [6]. Historically, the major primary function of 17β estradiol (E_2_) was the development of female secondary sexual characteristics and regulation of reproductive function. Today it is recognized that E_2 _exerts some effect on almost every organ in the body [7]. The effects of E_2 _and other estrogens have expanded to include roles in neurological function [8], retinal degenerative disease [9], cardiovascular health [10], and even sleep regulation [11].

Given the well documented mitogenic and possible genotoxic nature of endogenous estrogens and EMs [2,12,13], potential involvement of EMs in the carcinogenesis of an even greater variety of human tumors is conceivable. For example, some studies have suggested that estrogen may be involved in the development of skin cancer as skin keratinocytes possess estrogen receptors (ERs) [14], and oral contraceptives and hormone therapy decrease acne [15] and skin aging [16], respectively. Epidemiological studies examining associations between hormone therapy and melanoma risk have not been entirely conclusive, although some studies have shown a link between hormone use and increased risk of melanoma [17,18]. Epidemiologically, estrogens have also been linked to colon cancer, as men are more likely to develop this disease and hormone replacement therapy has been shown to reduce the risk of this cancer in women [19].

Owing to the pervasive nature of the functions of EMs, their effect on cancers may be more pronounced than previously thought. Determining EM roles in various cancers requires a lot of information, including tumor receptor status, aromatase activities, and the levels of these compounds within cells. Towards this aim, we systemically surveyed human tumor cell EM levels by using NCI-60 human tumor cell lines, including human breast, central nervous system (CNS), colon, ovarian, prostate, kidney, melanoma, leukemia, and non-small cell lung cancers. Detailed EM profiles in NCI-60 human tumor cell lines are summarized and reported in this manuscript.

## Materials and methods

### Reagents and materials

Cell pellets from NCI-60 human tumor cell lines were obtained from Developmental Therapeutics Program, NCI/NIH. Fifteen estrogens and EMs, including estrone (E_1_), estradiol (E_2_), estriol (E_3_), 16-epiestriol (16-epiE_3_), 17-epiestriol (17-epiE_3_), 16-ketoestradiol (16-ketoE_2_), 16α-hydroxyestrone (16α-OHE_1_), 2-methoxyestrone (2-MeOE_1_), 4-methoxyestrone (4-MeOE_1_), 2-hydroxyestrone-3-methyl ether (3-MeOE_1_), 2-methoxyestradiol (2-MeOE_2_), 4-methoxyestradiol (4-MeOE_2_), 2-hydroxyestrone (2-OHE_1_), 4-hydroxyestrone (4-OHE_1_), and 2-hydroxyestradiol (2-OHE_2_) were obtained from Steraloids, Inc. (Newport, RI, USA). Stable isotope-labeled estrogens (SI-EM), including estradiol-13,14,15,16,17,18-^13^C_6 _(^13^C_6_-E_2_) and estrone-13,14,15,16,17,18-^13^C_6 _(^13^C_6_-E_1_) were purchased from Cambridge Isotope Laboratories, Inc. (Andover, MA, USA); estriol-2,4,17-*d*_3 _(d_3_-E_3_), 2-hydroxyestradiol-1,4,16,16,17-*d*_5 _(d_5_-2-OHE_2_), and 2-methoxyestradiol-1,4,16,16,17-*d*_5 _(d_5_-2-MeOE_2_), were obtained from C/D/N Isotopes, Inc. (Pointe-Claire, Quebec, Canada). 16-Epiestriol-2,4,16-*d*_3 _(d_3_-16-epiE_3_) was purchased from Medical Isotopes, Inc. (Pelham, NH, USA). All EM and SI-EM analytical standards have reported chemical and isotopic purity ≥98%, and were used without further purification. Dichloromethane, methanol and formic acid were obtained from EM Science (Gibbstown, NJ, USA). Glacial acetic acid, sodium bicarbonate, and L-ascorbic acid were purchased from JT Baker (Phillipsburg, NJ, USA) and sodium hydroxide and sodium acetate were purchased from Fisher Scientific (Fair Lawn, NJ, USA). Dansyl chloride and acetone were purchased from Aldrich Chemical Co. (Milwaukee, WI, USA). All chemicals and solvents used in this study were HPLC or reagent grade unless otherwise noted.

### Preparation of stock and working standard solutions and calibration standards

Stock solutions of EMs and SI-EMs were each prepared at 80 μg/ml by dissolving 2 mg of the estrogen powders in methanol with 0.1% L-ascorbic acid to a final volume of 25 ml in a volumetric flask. The stock solutions are stable for at least two months while stored at -20°C. Stock solutions were analyzed at the beginning of each analysis to verify no time-dependent degradation of the EM and SI-EM standards had occurred. Working standards of EMs and SI-EMs at 8 ng/ml were prepared by dilutions of the stock solutions using methanol with 0.1% L-ascorbic acid.

MCF-10A cell lysate with no detectable levels of EMs was employed for preparation of calibration standards and quality control samples. Each calibration standard contained lysate from approximately 50,000 MCF-10A cells and was prepared by adding 2 μl of the SI-EM working internal standard solution (16 pg of each SI-EM) to various volumes of the EM working standard solution. These calibration standards typically contain 0.2 to 200 pg of each EM in 0.5 ml of MCF-10A cell lysate and were assayed in duplicate. The calibration standards cover three orders of magnitude.

### Sample preparation procedure

Samples were prepared and analyzed following a previously published method [20,21]. Briefly, each tumor cell pellet contained approximately 1 million cells. They were first suspended in 2 ml ice cold 12.5 mM NH_4_HCO_3 _solution. Cell lyses were prepared by tip sonication on ice in five cycles of 10-second pulses and 10-second breaks followed by 30-minute water bath sonication. To 0.5 ml of each cell lysate, 0.5 ml of freshly prepared 0.15 M sodium acetate buffer (pH 4.6) containing 16 pg of each SI-EM and 2 mg of L-ascorbic acid was added. Samples then underwent slow inverse extraction at 8 rpm (RKVSD™, ATR, Inc., Laurel, MD, USA) with 5 ml dichloromethane for 30 minutes. After extraction, the organic solvent portion was transferred into a clean glass tube and evaporated to dryness at 60°C under nitrogen gas (Reacti-Vap III™, Pierce, Rockford, IL, USA).

To each dried sample, 32 μl of 0.1 M sodium acetate buffer (pH at 9.0) and 32 μl of dansyl chloride solution (1 mg/ml in acetone) were added. After vortexing, the sample was heated at 60°C (Reacti-Therm III™ Heating Module, Pierce, Rockford, IL, USA) for 10 minutes to form the EM and SI-EM dansyl derivatives (EM-Dansyl and SI-EM-Dansyl, respectively). Calibration standards and quality control samples were hydrolyzed, extracted, and derivatized following the same procedure used for unknown cell samples. After derivatization, all samples were analyzed by capillary liquid chromatography (LC)-tandem mass spectrometry (MS^2^).

### Liquid chromatography-tandem mass spectrometry

LC-MS^2 ^analysis was performed using an Agilent 1200 series nanoflow LC system (Agilent Technologies, Palo Alto, CA, USA) coupled to a TSQ™ Quantum Ultra triple quadrupole mass spectrometer (Thermo Electron, San Jose, CA, USA). The LC separation was carried out on a 150 mm long × 300 μm internal diameter column packed with 4 μm Synergi Hydro-RP particles (Phenomenex, Torrance, CA, USA) and maintained at 40°C. A total of 8 μl of each sample was injected onto the column. The mobile phase, operating at a flow rate of 4 μl/minute, consists of methanol as solvent A and 0.1% (v/v) formic acid in water as solvent B. A linear gradient from 72 to 85% solvent B in 75 minutes was employed for separation of EMs and SI-EMs. The mass spectrometry conditions were: source, ESI; ion polarity,positive; spray voltage, 3200 V; sheath and auxiliary gas, nitrogen; sheath gas pressure, 10 arbitrary units; ion transfer capillary temperature, 270 °C; scan type, selected reaction monitoring; collision gas, argon; collision gas pressure, 1.5 mTorr; scan width, 0.7 u; scan time, 0.30 s; Q1 peak width, 0.70 u full-width half-maximum (FWHM); Q3 peak width, 0.70 u FWHM. The optimized selected reaction monitoring conditions for the protonated molecules [MH]^+ ^of EM-Dansyl and SI-EM-Dansyl were similar to those previously described [9,10].

### Quantification of estrogen metabolites

Quantification of EMs was carried out using Xcalibur™ Quan Browser (Thermo Electron) as previously described [20,21]. Briefly, calibration curves for the each EM were constructed by plotting EM-Dansyl/SI-EM-Dansyl peak area ratios obtained from calibration standards versus amounts of the EM injected on the column and fitting these data using linear regression with 1/X weighting. The amounts of EMs in cells were then interpolated using this linear function. Based on their similarity of structures and retention times, ^13^C_6_-E_2 _was used as the internal standard for E_2_; ^13^C_6_-E_1 _for E_1_; d_3_-E_3 _for E_3_, 16-ketoE_2_, and 16α-OHE_1_; d_3_-16-epiE_3 _for 16-epiE_3 _and 17-epiE_3_; d_5_-2-MeOE_2 _for 2-MeOE_2_, 4-MeOE_2_, 2-MeOE_1_, 4-MeOE_1_, and 3-MeOE_1_; d_5_-2-OHE_2 _for 2-OHE_2_, 2-OHE_1_, and 4-OHE_1_.

## Results and discussion

**Figure 1:**
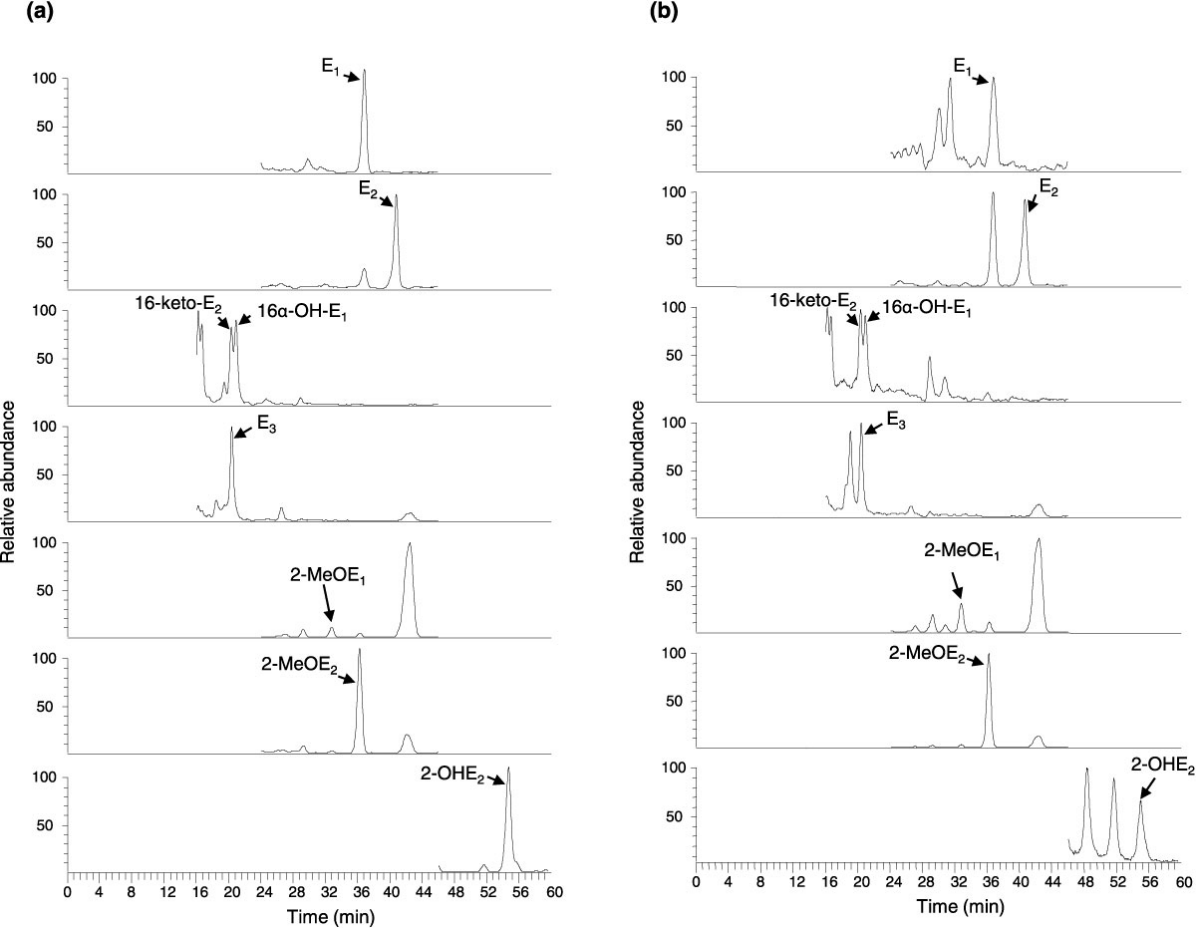
**Chromatograms showing the eight quantified endogenous estrogen metabolites for (a) the ovarian cancer cell line SK-OV-3 and (b) the colon cancer cell line HCC-2998**. 16-ketoE_2_, 16-ketoestradiol; 16α-OHE_1_, 16α-hydroxyestrone; 2-MeOE_1_, 2-methoxyestrone; 2-MeOE_2_, 2-methoxyestradiol; 2-OHE_2_, 2-hydroxyestradiol; E_1_, estrone; E_2_, estradiol; E_3_, estriol.

The levels of endogenous estrogens and EMs were measured in the NCI-60 cell lines, which comprise breast (n = 5), CNS (n = 6), colon (n = 7), leukemia (n = 6), melanoma (n = 9), non-small cell lung (n = 9), ovarian (n = 9), prostate (n = 2), and renal (n = 8) cancers. This study focused on measuring only the unconjugated, active forms of the EMs. Glucoronidated and sulfated forms of the EMs were not included in the analysis. All NCI-60 human tumor cell lines showed significant levels of E_1_, E_2_, 16-ketoE_2_, 16α-OHE_1_, E_3_, 2-MeOHE_1_, 2-MeOHE_2_, and 2-OHE_1_. The chromatograms showing the eight quantified endogenous EMs for an ovarian (SK-OV-3) and colon cancer cell line (HCC-2998) are shown in Figure 1. The peaks were generally well resolved and had good signal-to-noise ratios for all cell lines analyzed. While undetectable in all the others, 2-OHE_1 _was found in the non-small cell lung cancer cell line NCI-H460.

Within the same type of cancer, different tumor cell lines had substantially different levels of EMs (Table 1). For example, SF-539 and SNB-75 cells produced greater amounts of estrogens than the other CNS lines tested. HCC-2998 colon cancer cells, RMPI-8226 leukemia cells, SK-MEL-28, UACC-257, UACC-62, MALME-3M melanoma cells, EKVX, NCI-H23, NCI-H226 non-small cell lung (NSCL) cancer cells, OVCAR-4, OVCAR-5, SK-OV-3 ovarian cancer cells, and CAKI-1 renal cancer cells all produced greater amounts of estrogens than the other cell lines within their category. Furthermore, estrogen levels in these tumor cell lines were substantially elevated and even exceeded the levels typically found in well characterized estrogen-dependent and ER-positive tumor cells such as MCF-7 and T-47D.

**Table 1:** Levels of unconjugated endogenous estrogens (picograms) found in NCI-60 cell lines

Cancer	Cell Line	E_3_	16αOHE_1_	16-epiE_3_	2MeOE_1_	2MeOE_2_	E_1_	E_2_	2OHE_1_	2OHE_2_	Total
Breast	T-47D	0.80	0.03	0.33	2.70	0.54	3.65	87.37	NF	7.30	102.72
		(0.07)	(0.00)	(0.04)	(0.34)	(0.10)	(0.06)	(2.69)	NF	(1.22)	(3.18)
											
Breast	Hs-578T	0.43	0.03	0.22	0.62	0.94	8.13	74.11	NF	5.86	90.35
		(0.06)	(0.00)	(0.05)	(0.07)	(0.17)	(0.98)	(9.94)	NF	(0.83)	(11.95)
											
Breast	BT-549	0.58	0.02	0.06	0.65	0.45	8.45	58.97	NF	6.03	75.22
		(0.10)	(0.00)	(0.03)	(0.10)	(0.02)	(1.20)	(11.51)	NF	(1.26)	(14.15)
											
Breast	MDA-MB-231	0.90	0.02	0.57	1.78	0.35	6.30	36.73	NF	2.55	49.21
		(0.07)	(0.00)	(0.05)	(0.24)	(0.05)	(0.37)	(2.41)	NF	(0.33)	(2.71)
											
Breast	MCF-7	0.40	0.04	0.28	2.12	5.38	7.70	80.75	NF	1.76	98.42
		(0.03)	(0.00)	(0.06)	(0.23)	(0.47)	(0.71)	(14.44)	NF	(0.06)	(13.04)
											
CNS	SF-295	0.24	0.04	0.12	0.93	0.81	6.79	41.51	NF	7.01	57.44
		(0.03)	(0.00)	(0.01)	(0.07)	(0.12)	(0.93)	(2.57)	NF	(1.03)	(3.25)
											
CNS	SF-539	0.82	0.11	NF	1.73	1.22	26.24	175.50	NF	27.11	232.78
		(0.10)	(0.02)	NF	(0.13)	(0.08)	(2.01)	(13.14)	NF	(2.78)	(14.43)
											
CNS	SF-268	0.35	0.04	0.31	0.43	0.26	2.69	24.68	NF	7.67	36.42
		(0.01)	(0.00)	(0.03)	(0.05)	(0.01)	(0.10)	(1.33)	NF	(0.96)	(2.21)
											
CNS	U251	0.35	0.29	0.21	1.19	0.31	4.54	41.06	NF	12.61	60.55
		(0.04)	(0.03)	(0.02)	(0.11)	(0.04)	(0.21)	(4.27)	NF	(1.22)	(5.01)
											
CNS	SNB-19	0.31	0.02	0.08	1.27	0.55	5.09	29.34	NF	3.63	40.29
		(0.05)	(0.00)	(0.01)	(0.14)	(0.09)	(0.64)	(2.31)	NF	(0.25)	(3.45)
											
CNS	SNB-75	0.30	0.04	0.10	1.46	0.56	13.15	94.94	NF	2.23	112.79
		(0.04)	(0.00)	(0.01)	(0.23)	(0.03)	(0.57)	(1.97)	NF	(0.24)	(1.80)
											
Colon	SW-620	0.75	0.08	0.28	1.70	1.39	0.44	1.31	NF	2.68	8.63
		(0.11)	(0.01)	(0.05)	(0.14)	(0.23)	(0.05)	(0.09)	NF	(0.31)	(0.61)
											
Colon	HCT-116	0.12	0.02	0.27	1.40	0.80	0.45	2.17	NF	0.33	5.58
		(0.02)	(0.00)	(0.04)	(0.06)	(0.08)	(0.04)	(0.13)	NF	(0.03)	(0.11)
											
Colon	COLO-205	0.33	0.07	0.19	0.57	0.76	1.12	50.44	NF	15.20	68.69
		(0.02)	(0.01)	(0.02)	(0.07)	(0.07)	(0.08)	(4.57)	NF	(0.77)	(5.22)
											
Colon	KM12	0.55	0.04	0.31	1.09	0.44	0.14	1.77	NF	0.73	5.06
		(0.06)	(0.00)	(0.04)	(0.10)	(0.09)	(0.01)	(0.12)	NF	(0.13)	(0.49)
											
Colon	HT29	0.51	0.03	0.51	1.12	0.28	1.90	30.71	NF	0.51	35.56
		(0.01)	(0.00)	(0.06)	(0.12)	(0.03)	(0.10)	(1.12)	NF	(0.05)	(0.90)
											
Colon	HCT-15	28.10	0.40	1.08	0.87	0.29	0.88	12.25	NF	3.75	47.62
		(1.54)	(0.06)	(0.04)	(0.09)	(0.03)	(0.10)	(1.22)	NF	(0.36)	(3.05)
											
Colon	HCC-2998	0.88	0.10	0.45	3.10	0.92	17.10	209.34	NF	4.29	236.18
		(0.05)	(0.01)	(0.07)	(0.05)	(0.17)	(0.08)	(13.95)	NF	(0.14)	(14.05)
											
Leukemia	HL-60	3.89	0.03	0.29	1.58	3.15	18.78	36.61	NF	16.50	80.83
		(0.02)	(0.00)	(0.01)	(0.11)	(0.10)	(2.39)	(2.70)	NF	(2.81)	(2.39)
											
Leukemia	CCRF-CEM	0.41	0.04	0.06	0.66	1.33	1.18	3.48	NF	1.16	8.31
		(0.07)	(0.00)	(0.02)	(0.04)	(0.10)	(0.12)	(0.50)	NF	(0.10)	(0.91)
											
Leukemia	K562	0.42	0.02	0.21	0.57	2.23	0.56	13.88	NF	1.16	33.14
		(0.07)	(0.00)	(0.04)	(0.06)	(0.07)	(0.06)	(1.75)	NF	(0.11)	(1.24)
											
Leukemia	MOLT-4	0.30	0.08	0.13	0.55	0.77	2.04	7.77	NF	0.75	12.37
		(0.04)	(0.01)	(0.01)	(0.04)	(0.07)	(0.37)	(1.14)	NF	(0.09)	(1.53)
											
Leukemia	RMPI-8226	0.93	0.11	0.59	2.04	3.60	182.06	753.45	NF	2.93	945.71
		(0.07)	(0.02)	(0.11)	(0.30)	(0.16)	(5.19)	(48.20)	NF	(0.22)	(52.18)
											
Leukemia	SR	4.67	0.06	0.80	3.24	1.45	6.67	26.39	NF	1.22	44.51
		(0.53)	(0.01)	(0.15)	(0.51)	(0.22)	(0.47)	(1.38)	NF	(0.19)	(2.00)
											
Melanoma	MDA-MB-435	0.37	0.22	0.09	0.61	3.65	1.77	14.24	NF	2.82	23.77
		(0.03)	(0.04)	(0.02)	(0.11)	(0.76)	(0.18)	(0.91)	NF	(0.32)	(1.05)
											
Melanoma	SK-MEL-28	0.82	0.05	0.27	3.83	5.41	21.50	146.59	NF	27.77	206.24
		(0.03)	(0.01)	(0.02)	(0.33)	(0.99)	(2.12)	(15.34)	NF	(2.17)	(16.78)
											
Melanoma	UACC-257	1.20	0.36	0.37	0.74	7.00	36.36	130.70	NF	2.50	179.23
		(0.23)	(0.07)	(0.05)	(0.10)	(0.78)	(2.41)	(4.37)	NF	(0.22)	(6.37)
											
Melanoma	LOX IMVI	0.93	0.07	0.44	1.33	1.01	0.35	12.10	NF	8.26	24.49
		(0.11)	(0.01)	(0.07)	(0.06)	(0.07)	(0.02)	(0.55)	NF	(0.64)	(0.50)
											
Melanoma	UACC-62	0.20	0.30	0.06	1.41	1.47	17.49	98.71	NF	1.62	121.27
		(0.03)	(0.04)	(0.01)	(0.06)	(0.25)	(0.21)	(4.61)	NF	(0.30)	(4.82)
											
Melanoma	SK-MEL-2	1.56	1.60	0.38	2.21	0.62	6.66	50.93	NF	2.90	66.86
		(0.15)	(0.08)	(0.07)	(0.05)	(0.08)	(0.40)	(5.84)	NF	(0.33)	(6.40)
											
Melanoma	SK-MEL-5	0.38	0.38	0.13	14.84	0.52	11.64	72.02	NF	3.83	103.75
		(0.03)	(0.05)	(0.02)	(0.47)	(0.08)	(0.31)	(4.06)	NF	(0.50)	(5.25)
											
Melanoma	MALME-3M	0.36	0.06	0.32	3.88	0.45	1.26	94.27	NF	1.30	101.90
		(0.03)	(0.01)	(0.05)	(0.26)	(0.06)	(0.05)	(0.88)	NF	(0.13)	(1.13)
											
Melanoma	M14	1.33	0.05	0.18	1.24	0.69	1.21	13.95	NF	1.90	20.55
		(0.06)	(0.00)	(0.02)	(0.11)	(0.11)	(0.09)	(0.50)	NF	(0.28)	(0.91)
											
NSCL	A549	0.56	0.04	0.54	1.04	1.18	2.95	17.01	NF	2.19	25.51
		(0.06)	(0.00)	(0.05)	(0.05)	(0.07)	(0.27)	(0.86)	NF	(0.24)	(1.18)
											
NSCL	EKVX	1.70	0.31	0.92	1.10	7.16	15.16	125.95	NF	4.53	156.83
		(0.06)	(0.05)	(0.01)	(0.12)	(0.72)	(1.13)	(13.92)	NF	(0.47)	(12.21)
											
NSCL	HOP-62	1.48	0.08	0.78	2.37	4.70	6.19	25.45	NF	13.29	54.33
		(0.24)	(0.01)	(0.14)	(0.40)	(0.44)	(1.18)	(1.85)	NF	(1.56)	(5.04)
											
NSCL	NCI-H23	1.08	0.29	0.39	6.10	0.92	8.03	90.47	NF	15.79	123.07
		(0.05)	(0.04)	(0.08)	(0.43)	(0.05)	(0.64)	(2.81)	NF	(2.87)	(3.46)
											
NSCL	NCI-H460	0.18	0.05	0.15	1.36	1.15	4.03	59.76	27.89	2.21	96.78
		(0.01)	(0.01)	(0.00)	(0.23)	(0.04)	(0.06)	(3.33)	(0.32)	(0.12)	(3.69)
											
NSCL	NCI-H226	0.29	0.07	0.13	0.50	0.61	25.40	181.13	NF	21.63	229.75
		(0.02)	(0.01)	(0.01)	(0.02)	(0.07)	(1.05)	(7.48)	NF	(0.69)	(8.87)
											
NSCL	HOP-92	0.33	0.03	0.43	0.55	0.24	4.87	34.58	NF	2.20	43.24
		(0.01)	(0.00)	(0.08)	(0.03)	(0.04)	(0.28)	(1.51)	NF	(0.24)	(1.43)
											
NSCL	NCI-H522	0.18	0.03	0.06	1.38	0.31	4.25	32.36	NF	1.55	40.12
		(0.02)	(0.00)	(0.01)	(0.08)	(0.04)	(0.36)	(2.39)	NF	(0.31)	(2.58)
											
NSCL	NCI-H322M	0.19	0.04	0.13	0.80	0.45	0.72	7.01	NF	1.29	10.62
		(0.02)	(0.00)	(0.01)	(0.06)	(0.04)	(0.08)	(0.27)	NF	(0.12)	(0.13)
											
Ovarian	OVCAR-3	0.50	0.07	0.34	0.97	3.70	5.37	26.60	NF	5.47	43.02
		(0.05)	(0.01)	(0.04)	(0.09)	(0.54)	(0.73)	(1.63)	NF	(0.81)	(3.21)
											
Ovarian	OVCAR-5	0.16	0.03	0.13	1.26	0.33	12.85	107.47	NF	1.57	123.79
		(0.00)	(0.00)	(0.02)	(0.17)	(0.05)	(0.33)	(5.55)	NF	(0.14)	(5.89)
											
Ovarian	IGR-OV1	0.15	0.04	0.11	0.73	2.54	4.00	29.72	NF	0.66	37.94
		(0.01)	(0.00)	(0.00)	(0.01)	(0.36)	(0.15)	(0.70)	NF	(0.06)	(1.10)
											
Ovarian	NCI/ADR-RES	1.33	0.15	0.44	2.54	2.92	10.46	75.10	NF	4.61	97.56
		(0.08)	(0.02)	(0.04)	(0.28)	(0.14)	(0.31)	(0.47)	NF	(0.28)	(0.06)
											
Ovarian	SK-OV-3	1.78	0.13	0.39	0.73	0.55	12.26	132.33	NF	4.18	152.36
		(0.23)	(0.02)	(0.03)	(0.09)	(0.10)	(0.25)	(9.43)	NF	(0.63)	(9.94)
											
Ovarian	OVCAR-8	0.44	0.13	0.15	2.10	0.35	5.12	38.83	NF	3.61	50.73
		(0.02)	(0.03)	(0.03)	(0.17)	(0.06)	(0.22)	(1.01)	NF	(0.58)	(1.56)
											
Ovarian	OVCAR-4	0.43	0.06	0.31	3.90	0.43	19.79	139.32	NF	4.69	168.93
		(0.02)	(0.01)	(0.02)	(0.06)	(0.07)	(1.06)	(2.84)	NF	(0.46)	(2.82)
											
Prostate	PC-3	0.15	0.03	0.20	1.05	2.33	7.63	41.94	NF	0.63	53.96
		(0.02)	(0.00)	(0.03)	(0.05)	(0.14)	(0.56)	(2.30)	NF	(0.07)	(2.90)
											
Prostate	DU145	0.55	0.04	0.42	2.22	0.71	4.94	27.78	NF	2.55	39.22
		(0.05)	(0.00)	(0.05)	(0.16)	(0.10)	(0.29)	(1.02)	NF	(0.23)	(1.06)
											
Renal	UO-31	0.30	0.13	0.33	1.36	1.66	5.05	22.94	NF	2.45	34.22
		(0.03	(0.02	(0.02	(0.26	(0.26	(0.60	(1.18	NF	(0.15	(0.92
											
Renal	786-0	0.15	0.02	0.11	0.58	0.16	2.11	23.40	NF	1.54	28.07
		(0.02)	(0.00)	(0.01)	(0.05)	(0.02)	(0.16)	(0.14)	NF	(0.19)	(0.17)
											
Renal	SN12C	0.14	0.05	0.27	6.47	1.92	4.02	25.50	NF	0.89	39.25
		(0.01)	(0.01)	(0.02)	(0.29)	(0.17)	(0.07)	(0.97)	NF	(0.05)	(0.96)
											
Renal	CAKI-1	0.54	0.16	0.13	4.76	0.49	7.57	81.60	NF	6.52	101.77
		(0.05)	(0.03)	(0.03)	(0.42)	(0.07)	(0.61)	(1.42)	NF	(0.79)	(2.29)
											
Renal	RXF-393	0.27	0.03	0.09	0.47	0.44	0.34	14.48	NF	1.41	17.54
		(0.04)	(0.00)	(0.02)	(0.09)	(0.04)	(0.04)	(0.39)	NF	(0.13)	(0.38)
											
Renal	TK-10	0.65	0.25	0.42	2.54	0.58	7.87	37.29	NF	6.25	55.86
		(0.06)	(0.04)	(0.02)	(0.24)	(0.06)	(0.22)	(3.96)	NF	(0.60)	(4.27)
											
Renal	A498	0.53	0.02	0.16	0.29	0.50	3.05	37.53	NF	2.58	44.65
		(0.04)	(0.00)	(0.00)	(0.03)	(0.07)	(0.29)	(1.09)	NF	(0.37)	(1.45)
											
Renal	ACHN	0.43	0.08	0.19	1.25	0.39	6.17	39.27	NF	3.40	51.17
		(0.06)	(0.01)	(0.02)	(0.16)	(0.06)	(0.74)	(1.96)	NF	(0.28)	(2.51)

Data are expressed as mean (standard deviation) of three replicated analyses of 10^6 ^cells. E_3_, estriol; 16αOHE_1_, 16α-hydroxyestrone; 16-epiE_3_, 16-epiestriol; 2MeOE_1_, 2-methoxyestrone; 2MeOE_2_, 2-methoxyestradiol; E_1_, estrone; E_2_, 17β-estradiol; 2OHE_1_, 2-hydroxyestrone; 2OHE_2_, 2-hydroxyestradiol; CNS, central nervous system; NF, not found; NSCLC, non-small cell lung carcinoma.

Within each tumor cell line, E_2 _was by far the most abundant unconjugated estrogen followed by E_1 _and 2-OHE_2 _(Table 1). For the five breast cancer cell lines, E_2 _represented 75 to 85% of the total amount of unconjugated EMs measured. For six of the ovarian cancer cell lines, E_2 _represented 77 to 87% of the total estrogen content, while this percentage was only 62% for OVCAR-3 cells. T-47D and MCF-7 cells are both estrogen-dependent and ER-positive human breast cancer cells and have E_2 _levels at 87 and 81 pg/10^6 ^cells, which accounted for 85% and 82% of their total unconjugated estrogens, respectively. MDA-MB-231 is an estrogen-independent, ER-negative, HER2-positive human breast cancer cell line and still has E_2 _levels of about 37 pg/10^6 ^cells, which accounts for about 75% of its total unconjugated estrogen levels.

**Figure 2:**
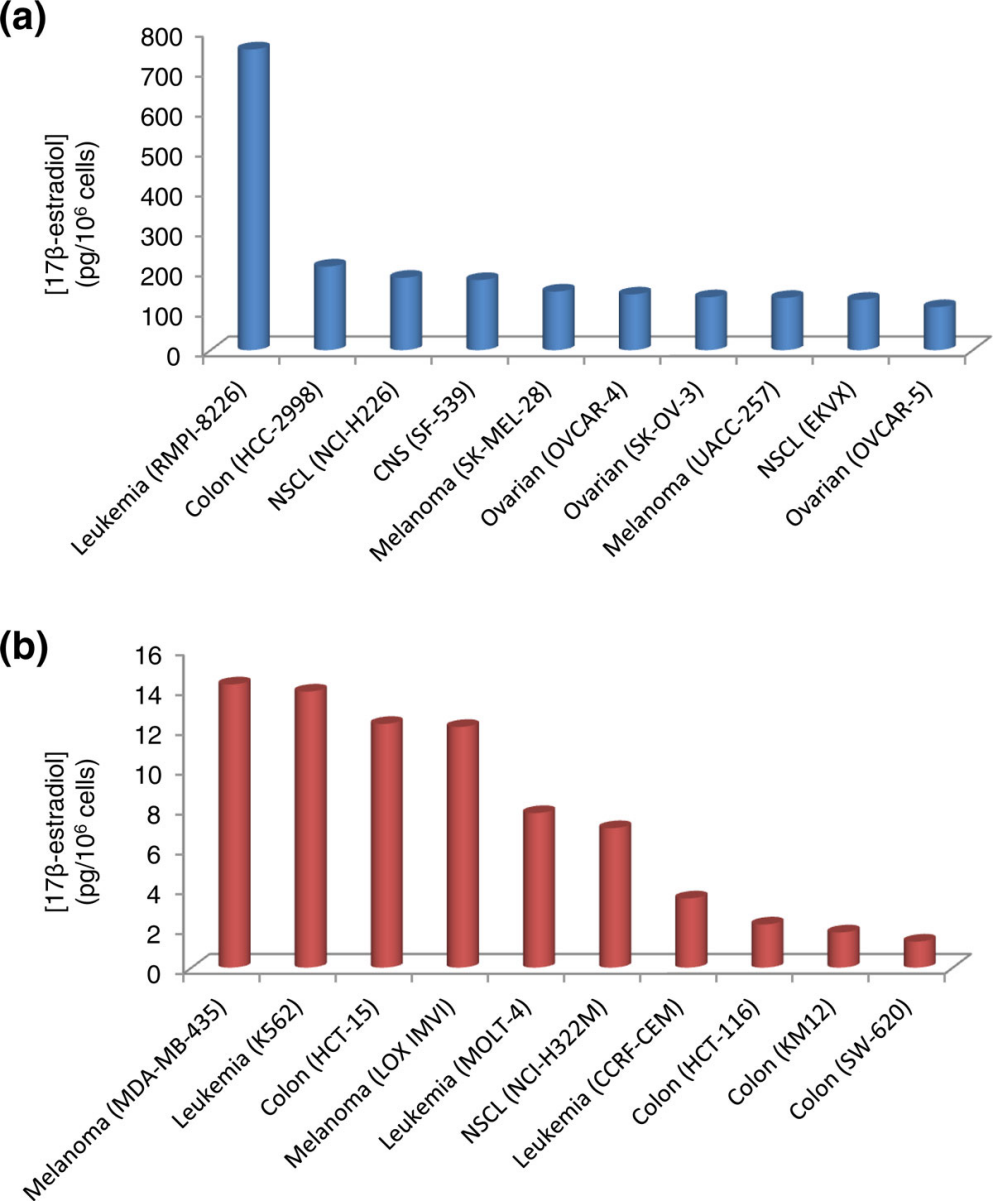
**Cell lines containing the highest and lowest 17β-estradiol (E_2_) levels within the NCI-60 panel**. **(a) **Cell lines with the highest E_2 _levels; **(b) **cell lines with the lowest E_2 _levels.

The cell lines with the highest E_2 _levels are shown in Figure 2a. Although estrogens are commonly associated with breast cancer, none of these cell lines were among those that contained the highest levels of E_2_. Consistent with evidence linking estrogen levels with cancers of the reproductive system in general, three ovarian cancer cell lines (OVCAR-4, OVCAR-5, and SK-OV-3) were amongst those with the highest E_2 _levels. OVCAR-4 and -5 are both ERα-negative, ERβ-positive ovarian cell lines who's growth is insensitive to E_2 _treatment [22]. While SK-OV-3 cells do express ERα, their growth is also insensitive to treatment with E_2 _[22]. The leukemia cell line RMPI-8226 possessed the highest levels of E_2 _(753 pg/10^6 ^cells). In fact, its E_2 _levels were more than 3.5-fold higher than the colon cell line HCC-2998, which contained the next highest level of E2 (209 pg/10^6 ^cells). This result correlates with a previous study showing that RMPI-8226 cells possess the highest ER levels compared to other leukemia and myeloid cell lines tested [23]. Previous studies have shown that the HL60 leukemia cell line possesses ERs and its proliferation is sensitive to E_2 _treatment. When the cells are maintained in a medium containing physiological concentrations (10^-9 ^M, 10^-8 ^M, 10^-7 ^M) of E_2_, cell growth is stimulated; however, pharmacological concentrations (10^-6 ^M) of E_2 _inhibit their growth [24]. Adding tamoxifen inhibited the stimulating effect of the estrogens by binding to and blocking the ER. The effect of estrogen was therefore associated with the presence of ERs in the human leukemic cell line HL60 and may be important in the proliferation of other leukemic cell lines.

Cell lines with the lowest E_2 _levels are shown in Figure 2b. Four of these were colon cell lines (HCT-116, HCT-15, KM12, and SW-620). Their E_2 _levels ranged from 1.31 to 12.25 pg/10^6 ^cells. To put into perspective the range of E_2 _values found in all the cell lines, SW-620 colon cells contained almost 575-fold less E_2 _than RMPI-8226 cells. The finding that colon cell lines generally contain low levels of E_2 _is consistent with a previous study that found ERs are present in colorectal tumors and human colonic cancer cell lines at very low levels [25].

**Figure 3:**
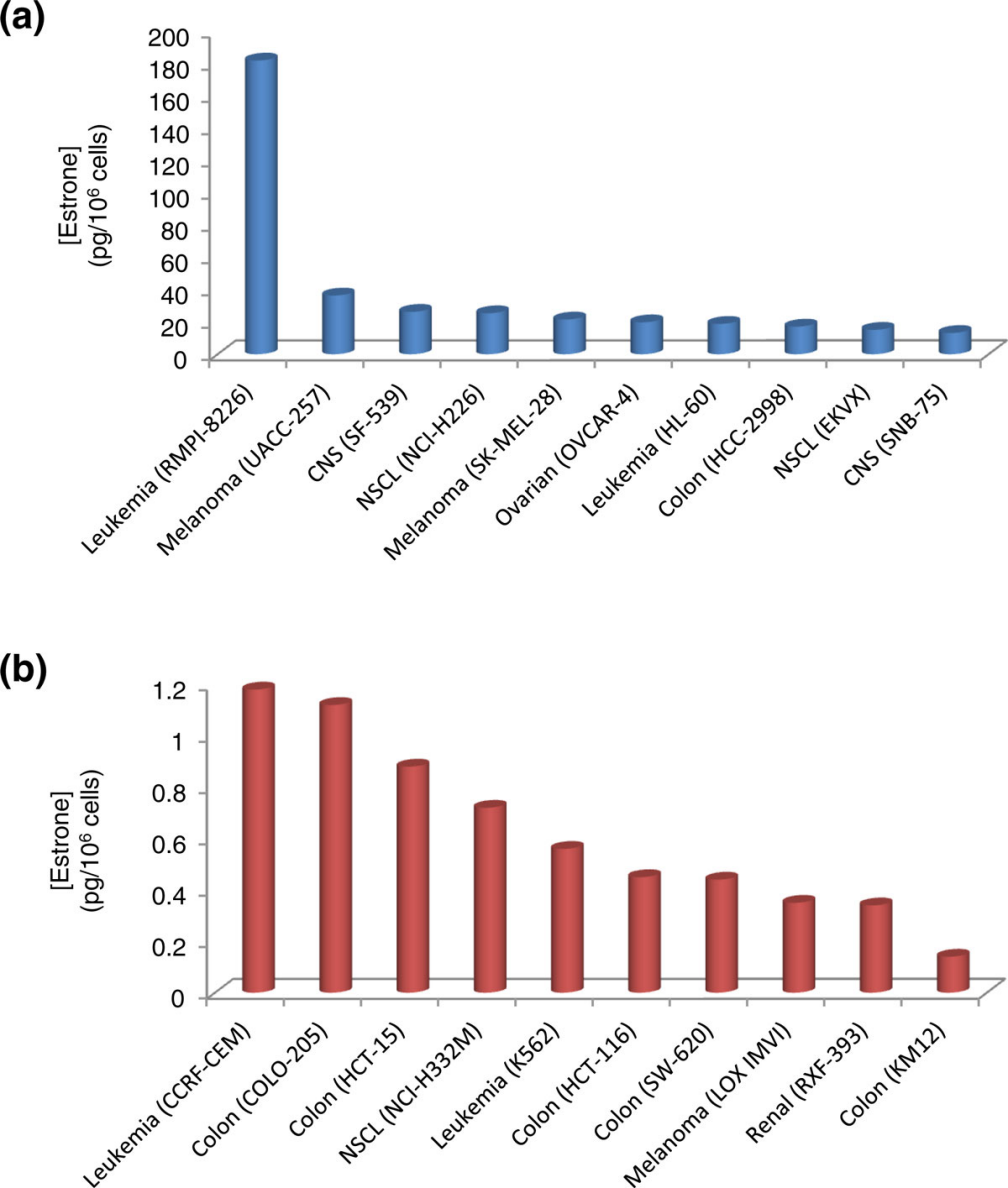
**Cell lines containing the highest and lowest estrone (E_1_) levels within the NCI-60 panel**. **(a) **Cell lines with the highest E_1 _levels; **(b) **cell lines with the lowest E_1 _levels.

As with E_2_, the leukemic cell line RPMI-8226 contained the highest levels of E_1 _of the NCI-60 cell lines (Figure 3a). The amount measured in this cell line was more than five-fold higher than that found in the cell line (melanoma UACC-257) containing the next highest levels of E_1_. Again, none of the five breast cancer cell lines tested was amongst the top ten E_1_-containing cells. The ovarian cancer cell line OVCAR-4 (19.79 pg/10^6 ^cells) was sixth on the list of cell lines containing the most E_1_. Two non-small cell lung carcinoma cell lines (EKVX and NCI-H226) were amongst the top ten in both E_1 _and E_2 _levels. This result is interesting considering that females that never smoked are far more likely to develop lung carcinoma than never-smoked males, suggesting a gender difference exists in the clinical and pathophysiology of lung cancer [21]. Recent studies showing aromatase-dependent synthesis of estrogens *in situ *in male and female lung cancers suggest that estrogens may contribute to the manifestation and progression of lung carcinoma [26-28].

Cell lines with the lowest E_1 _levels are shown in Figure 3b. COLO-205 was included along with the four colon cancer cell lines that were amongst the ten containing the lowest E_1 _levels (HCT-116, HCT-15, KM12, and SW-620). Their E_1 _levels ranged from 0.14 to 1.12 pg/10^6 ^cells. The levels of E_1 _found in KM12 colon cancer cells was approximately 1,300-fold less than that found in RPMI-8226 leukemia cells. Two leukemia cell lines, CCRF-CEM and K562, which were amongst those possessing the lowest E_2 _levels, also contained low E_1 _levels.

To identify general trends within the various cell lines tested, the means and standard deviations (SDs) of the total EM levels found in the cell types analyzed. As shown in Table 2, the leukemia cell lines had the highest overall mean total EM values (187.5 pg/10^6 ^cells). This value was almost twice as high as the cell types with the next highest total EM levels. Ovarian (96.33 pg/10^6 ^cells) and breast (83.18 pg/10^6 ^cells) cancer cells, which are commonly associated with estrogens, contained the second and sixth highest levels of total EMs. In fact, five of the cancer cell lines (ovarian, melanoma, CNS, NSCL, and breast) had total EM levels between 80 and 100 pg/10^6 ^cells. A noticeable feature of Table 2 are the very high SDs, but in particular that for the leukemia cell lines. To further explore what contributed to this high SD, the highest and lowest total EM levels measured for the individual cell lines in the different cell types were eliminated and the mean and SD values were recalculated. The mean EM levels for the leukemia cell lines after eliminating the cell lines with the highest and lowest concentrations was 42.71 pg/10^6 ^cells with a SD of 28.68; dropping their overall rank from first to sixth. The cell types having the three highest EM levels after eliminating the cell lines with the highest and lowest concentrations were (in order) ovarian (93.29 pg/10^6 ^cells, SD = 46.84), melanoma (88.75 pg/10^6 ^cells, SD = 55.53), and breast (88.00 pg/10^6 ^cells, SD = 11.78).

**Table 2:** Means and standard deviations of total estrogen levels measured in cell types within the NCI-60 cell line panel

	Mean	SD	Mean (-high/low)*	SD (-high/low)*
Breast	83.18	21.70	88.00	11.78
CNS	90.05	75.07	67.77	31.31
Colon	58.19	82.12	33.21	26.64
Leukemia	187.48	372.4	42.71	28.67
Melanoma	94.23	67.69	88.75	55.49
NSCL	86.69	71.98	77.13	49.25
Ovarian	96.33	54.00	93.49	46.84
Prostate	46.59	10.42	NA	NA
Renal	46.57	25.49	42.20	10.44

Mean and standard deviation (SD) are presented for all values within specific cell types and for values after eliminating cell lines with the highest and lowest total estrogen levels. CNS, central nervous system; NSCLC, non-small cell lung carcinoma. *Mean and standard deviation calculated after removing cell lines containing highest and lowest total measured estrogen levels. NA, only two prostate cell lines were included in NCI-60 cell lines analyzed.

The finding that the melanoma cell lines contained relatively high levels of endogenous estrogen and EMs is interesting. Two melanoma cell lines in particular, SK-MEL-28 and UACC-257, were among those containing the highest levels of E_1 _(21.50 and 36.36 pg/10^6 ^cells, respectively) and E_2 _(146.59 and 130.70 pg/10^6 ^cells, respectively). Only four other cell lines, SF-539 (CNS), NCI-H226 (NSCL), RMPI-8226 (leukemia) and HCC-2998 (colon), contained higher levels of total estrogens. High-affinity E_2 _receptors have been reported for primary human melanomas [29] and patients expressing these receptors seem to have a better prognosis, suggesting E_2 _may inhibit the growth of these melanoma tumors [30]. While previous studies have identified the classical ER in only a small percentage of human melanomas via immunohistochemistry [30], the low affinity type II ER has been characterized in a variety of human melanomas [31]. This receptor has the same affinity as the classical receptor and also binds tamoxifen. Treating SK-Mel 23 melanoma cells with E_2 _has been shown to inhibit their growth, while pre-treating the cells with tamoxifen (an anti-estrogen) blocks the effects of E_2 _[32]. The prevalence of E_2 _within the melanoma cell lines may prevent uncontrolled cell proliferation by acting back upon the cells and binding to the type II ER.

In general, unconjugated E_3_, 16αOHE_1_, and 16-epiE_3 _were less abundant except for in HCT-15 colon tumor cells, which had a greater amount of E_3 _than E_2_. The catechol estrogen 2-OHE_2 _was the only catechol estrogen detected in the tumor cell lines, except for the NSCL cancer cell line NCI-H460, which also contains a relatively high level of 2-OHE_1 _(Table 1). No unconjugated 4-hydroxy catechol estrogens were detected in any of the NCI-60 tumor cells. This result is likely due to the fact that 4-hydroxy catechol estrogens are quickly transformed into other reactive species such as their quinones and semi-quinones, which could damage DNA and lead to tumor initiation [2,33,34]. In contrast, 2-hydroxy-catechol estrogens largely form stable conjugates such as 2-MeOHE_1 _and 2-MeOHE_2_. Significant levels of both of these EMs were found in all NCI-60 cell lines tested in this study.

This study measured the unconjugated levels of endogenous estrogens and EMs in the NCI-60 cell panel. From our previous experience, if we had measured the conjugated levels by adding a sulfatase/glucoronidase enzyme to deconjugate sulfated and glucoronidated molecules prior to LC-MS^2 ^analysis, we would expect to see a large increase in the levels of every metabolite that was routinely detected. We would also expect that 16-epiE_3_, 17-epiE_3_, 4-MeOE_1_, 3-MeOE_1_, 4-MeOE_2_, 2-OHE_1_, and 4-OHE_1 _would also be detectable. Our studies analyzing serum have shown that endogenous estrogens and EMs exist primarily (that is, 90%) in the conjugated forms in the circulation [20]. While this disparity between conjugated and unconjugated forms of these steroid hormones may not be as large in cells, we predict that a large amount of endogenous estrogens and EMs exist within cells in their conjugated forms. It is interesting to note that when we analyze serum, only E_1_, E_2_, E_3_, 2-MeOHE_1 _and 2-MeOHE_2 _are detected in their unconjugated forms [20]. In the NCI-60 cell lines we were able to also routinely detect 16-αOHE_1_, 16-epiE_3_, and 2-OHE_2_. Unfortunately, it is difficult to directly compare the estrogen and EM levels as the cell line concentrations are recorded in pg/10^6 ^cells, while those in serum are measured as pg/ml. The fact that more compounds are detected in their unconjugated forms in the cell lines, however, suggests that, in general, the concentrations of estrogens and EMs are higher in cells than in the circulation.

To determine if ER status correlates with the levels of estrogens and EMs identified in the various cell lines, we compare our data to that published by Holbeck *et al*. [35], who measured the mRNA levels of 48 nuclear receptors in 51 of the NCI-60 cell lines. The mRNA levels of ERα for nine of the cell lines that were found to contain the highest E_2 _levels were measured in this study. Of these, detectable ERα levels were found for SKOV-3, OVCAR-4, UACC-257, SK-MEL-28, and SF-539 cell lines. No ERα mRNA was detected for HCC-2998, NCI-H226, EKVX, and OVCAR-5 cell lines. The cell lines with the highest levels of ERα mRNA were SK-OV-3, and two breast cancer cell lines, MCF-7 and T-47D. Of these, only SK-OV-3 was among the cell lines containing the highest amounts of E_2_. We also compared the ERα and E_2 _levels within the nine melanoma cell lines analyzed in both studies. In this case, the six melanoma cell lines with detectable levels of ERα mRNA (SK-MEL-28, UACC-257, UACC-62, SK-MEL-2, SK-MEL-5, and MALME-3M) contained the highest amounts of E_2 _within that group. The melanoma cell lines containing the lowest E_2 _concentrations (M14, LOX IMVI, and MDA-MB-435) did not show detectable levels of ERα. Overall, there is no obvious correlation between ERα and E_2 _levels; however, only about 25% of the cell lines had detectable levels of ERα whereas E_2 _could be measured in every one.

## Conclusions

This study utilized an LC-MS^2 ^approach with the capability of measuring up to 15 different EMs to measure the levels of endogenous estrogens within the NCI-60 cell lines. Eight of the measured endogenous estrogens were consistently observed in all of the NCI-60 cell lines, providing an unprecedented view of these metabolites within these cancer cell lines. What is particularly striking is that the levels of EMs in well-known estrogen-dependent cancers such as ovarian and breast were not substantially greater than those found in other types of cancer cell lines. In fact, none of the breast cancer cell lines were amongst the top ten that contained the highest levels of E_1 _or E_2_. Cell lines not generally associated with estrogens, such as leukemia, colon, CNS, and NSCL, were found to have appreciable levels of these metabolites. The broad presence of EMs within the NCI-60 cell lines suggests that many cancers outside of the reproductive system may respond to treatments with anti-estrogens such as tamoxifen, toremifene, and fulvestrant. Considering technologies for measuring estrogen levels in biological samples is considerably improved, it is now worth the effort to test various tumors for the levels of these metabolites.

## Abbreviations

16-epiE_3_: 16-epiestriol; 16-ketoE_2_: 16-ketoestradiol; 16α-OHE_1_: 16α-hydroxyestrone; 17-epiE_3_: 17-epiestriol; 2-MeOE_1_: 2-methoxyestrone; 2-MeOE_2_: 2-methoxyestradiol; 2-OHE_1_: 2-hydroxyestrone; 2-OHE_2_: 2-hydroxyestradiol; 3-MeOE_1_: 2-hydroxyestrone-3-methyl ether; 4-MeOE_1_: 4-methoxyestrone; 4-MeOE_2_: 4-methoxyestradiol; 4-OHE_1_: 4-hydroxyestrone; CNS: central nervous system; E_1_: estrone; E_2_: estradiol; E_3_: estriol; EM: estrogen metabolite; ER: estrogen receptor; LC: liquid chromatography; MS^2^: tandem mass spectrometry; NSCL: non-small cell lung; SD: standard deviation; SI-EM: stable isotope-labeled estrogen. Bre: breast; Col: colon; Leu: leukemia; Mel: melanoma; Ovc: ovarian; Pros: prostate; Ren: renal.

## Competing interests

The authors declare that they have no competing interests.

## Authors' contributions

TV participated in the conception, design, and implementation of the experiments, the analysis and interpretation of the data and drafting of the manuscript. XX participated in the conception, design, and implementation of the experiments, conducted the sample preparation and data acquisition and participated in the analysis and interpretation of the data and drafting of the manuscript. All authors have read and approved the final version of the manuscript for publication.
